# John Snow and the contaminated water of River Ganga at Kumbh Mela in India

**DOI:** 10.1093/inthealth/ihaf081

**Published:** 2025-08-15

**Authors:** Sushila Tiwari, Rahul M Jindal, Dileep Mavalankar

**Affiliations:** Strayer University, 2121 15th St. N. in Arlington, VA, USA; Cybersecurity, Office of Human Rights, 21 Maryland Avenue, Rockville, Maryland 20850; Indian Institute of Public Health Gandhinagar, India; Indian Institute of Public Health Gandhinagar, Gandhinagar - 382042

**Keywords:** cholera, dysentery, fecal coliform counts, John Snow, Kumbh Mela ‘*Festival of the Sacred Pitcher*’, public health, typhoid

Dear Editor,

The banks of the Ganga, in the city of Prayagraj (previously called Allahabad), have drawn pilgrims from across the world for centuries, standing as a timeless symbol of spiritual devotion, and they host the Kumbh Mela ‘*Festival of the Sacred Pitcher*’, one of humanity's largest religious gatherings.^1^ The 2025 *Maha* (mega) Kumbh Mela, held from 13 January to 26 February 2025 in the historic city of Prayagraj, marked a remarkable achievement for India, drawing an estimated 600 million participants.^2^ While the Maha Kumbh 2025 demonstrated India's ability to organize a mass religious congregation of unparalleled scale, the tragic stampede on 9 February 2025 that claimed 30 lives and injured >60, exposed critical shortcomings in crowd management, at least on that day.^3^ It is possible that overprioritization of large numbers of national and international very important person devotees to the event may have diverted resources that could have been spent on sanitation and environment.^4^

Amidst the celebrations of the Maha Kumbh 2025, critical environmental considerations also came to the forefront. Data collected by the Central Pollution Control Board (CPCB) identified notable concerns regarding river water quality, highlighting the broader, systemic challenges associated with water resource management in India.

The CPCB conducted daily monitoring of water quality at five locations in Prayagraj and two sites further upstream on the Ganga River from 12 January 2025 to 4 February 2025. The CPCB's report, submitted to the National Green Tribunal (NGT), indicated high levels of fecal coliforms, rendering the river water unsafe for bathing during the Maha Kumbh.^3^ Elevated biochemical oxygen demand (BOD) levels, exceeding the standard of 3 mg/L, were reported, particularly on auspicious bathing days. On *Paush Purnima* (13 January), BOD levels exceeded permissible limits, while on *Makar Sankranti* (14 January), fecal coliform counts were more than fourfold above the threshold (11 000 MPN/100 mL). Measurements on 20 January reached 49 000 MPN/100 mL, 19 times the permissible limit.^5^

The NGT reprimanded the Uttar Pradesh Pollution Control Board (UPPCB) for allowing mass bathing in contaminated waters. The UPPCB maintained that the water quality largely met bathing standards, citing data collected before the festival began. However, discrepancies between agency reports and the absence of timely public health advisories revealed significant gaps in environmental and health risk communication during the event.^6^

While the CPCB already tracks water quality at specified intervals, the same is not necessarily communicated in real time or translated into timely advisories for the public. As a step towards greater environmental transparency and public health protection, public, real-time water-quality dashboards along the lines of air-quality indices and accessible via web and mobile platforms, as well as local physical sign boards, need to be established. Such measures would enable individuals to make informed decisions regarding ritual bathing or consumption of raw river water, which are considered very holy.

It is cavalier on the part of the central, state and district governments that an estimated 600 million people's health should have been put at risk when they bathed in such potentially polluted water. There was no special disease surveillance mechanism set up by any government agency to monitor cases of water-related diseases among the pilgrims traveling back to their place of residence.^7^ Physicians from some places in India had reported a surge in post-Kumbh Mela health conditions, varying from mild fungal skin infections to respiratory and gastrointestinal diseases.^8^ The Government of India did not produce any publicly available consolidated health, safety and environmental reports giving data on what happened during the whole event and what lessons can be learnt in future.

The Thames River in London was also heavily contaminated in the middle of the nineteenth century, which contributed to numerous cholera outbreaks. However, the work of the well-known public health physician, John Snow, demonstrated that cholera in London was caused by water contamination through his careful scientific investigation and observations on deaths due to cholera, and published his work in a book. After his work, all of the city's sewage was treated and diverted away from the river, cleaning up the Thames.^9^

The modern and aspirational India needs a clear and transparent system of monitoring river water quality, and making that data public so that people can decide if they want to bathe or drink raw river water. Faith in a religious belief is not a matter of debate here, but what is sad is that the 150-y-old science of water contamination and disease surveillance discovered by John Snow in London has not yet been implemented in Indian cities.

A high-powered body should be formed and laws already in existence should be stringently implemented by stakeholders, which include state governments, the National Centre for Disease Control, the Integrated Disease Surveillance Programme, the National Vector Borne Disease Control Programme Food Safety and Standards Authority, as well as international partners such as the Centers for Disease Control and Prevention and the WHO India country offices.^7^

In conclusion, medical, health, sanitation and crowd control data from the 2025 Maha Kumbh reinforces the urgent necessity for comprehensive social health measures in mass congregations.^10^ We recommend that a detailed report on health, safety and environment be published by the Government of India that will inform future mass gatherings.

## Data Availability

The data underlying this article are available in the article and in its online supplementary material.
